# Exploring the electrical and magnetic characteristics of novel barium-doped bismuth ferrite (Bi_0.9_Ba_0.1_FeO_3_) nanocomposites and their applications for electrocatalytic degradation of Congo red dye

**DOI:** 10.1039/d5ra00469a

**Published:** 2025-04-07

**Authors:** M. H. Ghozza, Tarek A. Yousef, Abdullah Al-Dakhil, Hela Ferjani, Abeer M. Alosaimi, Reda Abdel-Hameed, Elbadawy A. Kamoun, H. Y. Zahran, Ahmed T. Mosleh, I. S. Yahia

**Affiliations:** a Basic Science Department, Cairo Higher Institute for Engineering, Computer Science and Management New Cairo Egypt +20-1283320302; b Chemistry Department, College of Science, Imam Mohammad Ibn Saud Islamic University (IMSIU) Riyadh 11623 Saudi Arabia; c Center for Innovation and Entrepreneurship, Imam Mohammad Ibn Saud Islamic University (IMSIU) Riyadh 11623 Saudi Arabia; d Department of Chemistry, College of Science, Taif University P.O. Box 11099 Taif 21944 Saudi Arabia; e Basic Science Department, Preparatory Year, University of Hail Hail Saudi Arabia; f Department of Chemistry, College of Science, King Faisal University Al-Ahsa 31982 Saudi Arabia ekamoun@kfu.edu.sa badawykamoun@yahoo.com; g Laboratory of Nano-Smart Materials for Science and Technology (LNSMST), Department of Physics, Faculty of Science, King Khalid University PO Box 9004 Abha 61413 Saudi Arabia; h Nanotechnology Section, Egyptian Company for Carbon Materials El-Sheraton/El-Nozha Cairo 11757 Egypt

## Abstract

Bismuth perovskite Bi_0.9_Ba_0.1_FeO_3_ nanoparticles were synthesized by a solution-combustion technique at a fuel-to-oxidizer ratio equal to unity (*∅* = (F/O) = 1), where the effect of fuel type on their structural, electric, magnetic, and photocatalytic properties was discussed. Using Rietveld refinement with FullProf software, the prepared materials were characterized by XRD and SEM to examine their composition and morphology. Results revealed that the perovskite's pure phase ranged from 74% to 100%. Meanwhile, Scherrer, Williamson–Hall, and SEM investigations were used to calculate the crystallite sizes of the samples, which ranged from 18.5–27.7 nm, 23–32 nm, 23.8–34.3 nm, and 53.8–292.8 nm, respectively. In addition, the increase in DC conductivity is explained by decreased grain boundary scattering, due to the reduction of crystallite size. The multiferroic nanoparticles' estimated activation energy ranged from 0.39 to 0.07 eV. The transition temperature was 368 K for urea and triethanolamine (TEA) samples, while the other samples were pushed to a lower temperature, where conduction followed non-adiabatic small polaron hopping (SPH). Meanwhile, TEA and fuel-free samples appear to have a high magnetization parameter. The coercivity Hc of the TEA sample is three times greater than the others. According to the tests conducted to assess the nanoparticles' electrocatalytic performances, every fuel utilized in nanoparticle production process significantly impacts the electrocatalytic degradation of Congo red (CR) dye. When the 4 minutes experiment was over, all dye content in the solution was eliminated. The synthesized Bi_0.9_Ba_0.1_FeO_3_ using various fuels considerably impacts the parameters under study. Therefore, appropriate magnetic, electrical, and electrocatalysis properties were achieved by modifying the fuel type.

## Abstract 2

Chemical structure of Congo red dye.

## Graphical abstract

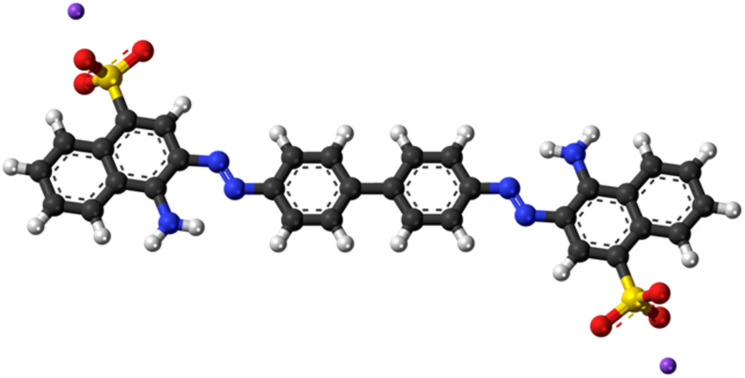

## 1 Introduction

Advanced nanomaterials based on bismuth ferrite perovskite are attracting considerable attention because of their intriguing physics and possible uses in fields like spintronics and data storage microelectronics.^1^ Due to the stereochemical activity of the Bi^3+^ ion electron pair, BiFeO_3_ is a rhombohedral distorted perovskite with the space group *R*3*c* and ferroelectric order (*T*_C_ ∼ 830 °C). BiFeO_3_ has a modulated cycloidal spin structure with a long periodicity of 62 nm and a G-type antiferromagnetic magnetic structure below *T*_N_ = 370 °C.^1^ It was difficult for several researchers to synthesize pure BiFeO_3_ since it is primarily contaminated with secondary phases such as Bi_2_O_3_ and Bi_2_Fe_4_O_9._^2–4^ Additionally, single BiFeO_3_ nanoparticles with divalent cations (Ba^2+^ and Ca^2+^) replacing the trivalent cations of Bi^3+^ have exhibited weak ferromagnetism at room temperature.^5^

Bismuth is very volatile since it only has one pair of electrons. BiFeO_3_'s commercial applications are limited due to its impurity and non-stoichiometry issues. Creating nanostructured materials that will cause the spin cycloid to break is one method to tackle these challenges, as long as the particle size is equal to the spin cycloid's, thus increasing the magnetism. Therefore, doping bismuth ferrite with different elements such as rare earth, alkaline earth metal, and transition metal in the A or B sites is another method to enhance its magnetoelectric qualities.^6^ Consequently, barium-doped bismuth ferrite nanostructures (Bi_0.9_Ba_0.1_FeO_3_) were prepared by auto-combustion using different solvent approaches. To create such a material with the appropriate stoichiometry, porosity, and phase composition, specific methods such as hydrothermal,^7^ sol–gel,^8^ co-precipitation,^9^ microwave irradiation, and solution-combustion have been reported previously.^10^ These methods necessitate extensive processing stages, lengthy durations, and complex apparatus. Simple metal oxides, mixed oxides, ferrites, perovskites, spinels, garnets, nanophosphors, phosphates and hydroxy-phosphates, metals and metal–ceramic composites, microspheres, and metal sulfides are just a few of the fundamental kinds of nanostructures that can be produced using the appealing solution-combustion synthesis method with comparatively less time and effort.^11^

The lives of humans and other living things are in danger due to pollution, which has become a serious issue with the recent industrial development.^12^ Several sources, including industrial discharge, sewage effluents, and agricultural industries, cause water contamination. Numerous dangerous and detrimental compounds, particularly organic pollutants like pesticides, antibiotics, and dyes, are present in contaminated water.^13^ To verify the effectiveness of treatment and the environment's safety, it is also crucial to conduct a chemical analysis of the target pollutants and degradation products found in treated wastewater and industrial effluents. Therefore, further research is required to determine the toxicity and quality of the treated wastewater using toxicity tests.^14^

Dyes are colorful materials used in various industrial products, including paper, textiles, leather, furniture, and cosmetics.^15,16^ Almost every industry uses dyestuffs to dye their products. Over 7 × 10^5^ tons and approximately 10 000 different dyes and pigments are produced worldwide annually.^17^ Approximately 10–15% of the dye is lost in the effluent during dyeing.^18^ Azo dyes comprise 50–70% of all dyes and are the main family of synthetic aromatic dyes. Congo red (CR) is an asymmetric, sulfonated azo dye that is a member of the protein-binding dye class [1-naphthalenesulfonic acid, 3,3′-(4,4′-biphenylene bis(azo))bis(4-amino-)disodium salt]. The hue of CR solution depends on its pH. CR is an anionic bis azo dye known to metabolize human carcinogens such as benzidine.^19^ The structural stability of these dyes makes them resistant to biodegradation due to which they are considered potential organic pollutants.^20^

Several methods have occasionally been used to exclude various dye types from effluents, including electrochemical coagulation, photocatalytic decolorization, electrochemical oxidation, adsorption, and microbiological degradation.^21–25^ Electrochemical methods have shown increased attention in wastewater cleanup throughout the last 10 years.^26^ They fall into one of the following categories for dye removal: indirect electro-oxidation with powerful oxidants, electro-oxidation with active chlorine, electrocoagulation, electrochemical reduction, and electrochemical oxidation.^27^ Since the electron, the primary reagent, is clean, these methods are environmentally friendly.^28^ They have benefits, including low operating temperatures, easy operation, and basic equipment.^29^ The high removal yields of contaminating chemicals with maximal energy resource management are noteworthy.^26^ On the surface of the electrodes, the electric current causes redox reactions that change and destroy the organic component and nearly oxidize it completely to CO_2_ and H_2_O.^30^

0ferrite nanostructures can be created using various techniques, including chemical vapor deposition and precipitation,^31^ electrochemical, microwave,^32^ sol–gel,^33^ Pechini method,^34^ and combustion^35^ Every technique has pros and cons of its own. When creating nanostructured BiFeO_3_, it is important to regulate the shape of the nanoparticles. Pure BiFeO_3_ synthesis has always been difficult since the final product is typically tainted with secondary phases like Bi_2_O_3_ and Bi_2_Fe_4_O_9_.^36^ Among these methods, auto-combustion methods are fast and easy, and this technique makes it feasible to manage the stoichiometry of chemical composition and dope different foreign atoms. An exothermic redox reaction between the precursor metal nitrates, regarded as an oxidant and a fuel, is essentially the solution-combustion synthesis. After all the water evaporates, the reactant mixture dehydrates into a gel and eventually self-ignites, either burning or smoldering. The reactants' self-combustion produces crystalline powder products with the appropriate stoichiometry.^37^ The temperature of calcination,^38,39^ the type and quantity of fuel (mixed or single),^39,40^ the fuel-to-oxidizer ratio (*∅*),^41^ metal precursors,^42^ and the reaction atmosphere, *e.g*. (air, N_2_, Ar, and He)^43^ all have a significant impact on the combusted product's crystallinity and particle size. Hence, in this study, Bi_0.9_Ba_0.1_FeO_3_ has been successfully produced by combustion methods using dissimilar fuels such as glycine without solvent, glycine with solvent, urea, glucose, TEA, and EDTA. Because urea was said to be the best fuel for metal nitrates in solution combustion synthesis, some authors^34^ favored it, while others preferred glycine and EDTA.^44–46^ Few previous studies reported variables *∅* and examined some of their outcomes. In the beginning, Deshpande *et al.*,^47^ focused on the effects of *∅* the fuel-nitrate system on iron oxide phase development and pore structure. In a few instances, they noticed the presence of several phases. Singh *et al.*, Toniolo *et al.*, and Ghosh used polyvinyl alcohol (PVA), urea, and glucose as the fuel with varying *∅*, respectively.^48–50^

**Fig. 1:**
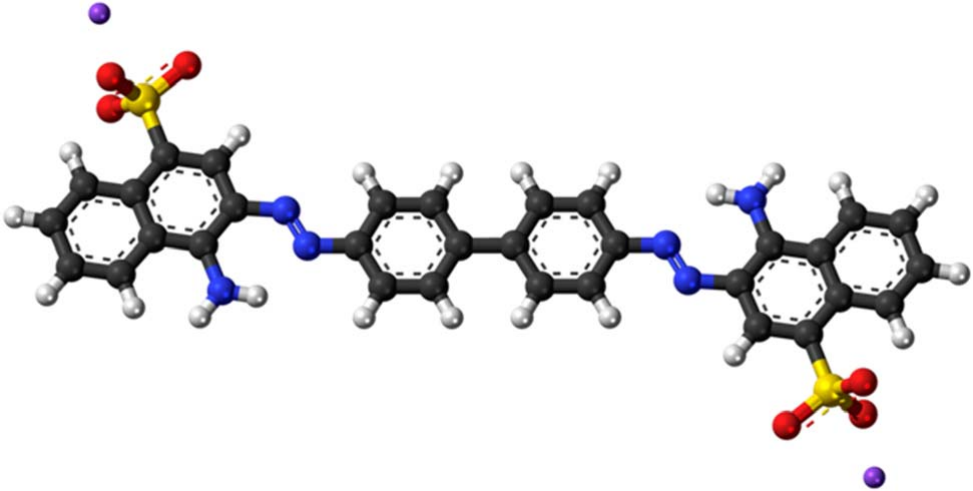
Structure of Congo red dye.^52^

Additionally, Fathi *et al.*^36^ found that iron oxide with *∅* = 1 had two different phases in various oxides and that glycine was the fuel that produced the highest saturation magnetization. Conversely, fuel-deficient iron oxide (*∅* = 0.7) had the maximum saturation magnetization, according to Wang *et al.*^51^ A different author looked into how *∅* (0.0, 0.5, 1.0, and 1.5) affected the electrochemical energy storage characteristics and found that *∅* = 1.0 performed the best. The magnetic and electric characteristics of BFO need to be improved for new electronic applications. Thus, examining this effect on the structure, electric and magnetic properties, and photocatalysis have been investigated and discussed in detail (Fig. 1).

To the best of our knowledge, we report here for the first time in the literature the formation of rhombohedral Bi_0.9_Ba_0.1_FO_3_ in a good perovskite phase ratio by various fuels and the obtained hard ferromagnetic-like properties that are observed and studied in detail. Moreover, Ba^2+^ dopant ions were chosen to replace trivalent Bi^3+^ cations in BiFeO_3_ with another divalent cation. For example, Ba^2+^ is expected to change the electrical and magnetic properties by reducing valence fluctuations in Bi^3+^. Previous research indicates that most published reports primarily examined the impact of differences *∅*. However, few studies have addressed the combustion technique of synthesis with various fuels and have not provided a thorough examination. Thus, in this study, we intend to examine the structural, electric, magnetic, and photocatalytic characteristics of Bi_0.9_Ba_0.1_FeO_3_ synthesized by a solution-combustion approach with fixed *∅* = 1.

## 2 Materials and methods

### 2.1. Material

Bismuth subnitrate Bi_5_H_9_N_4_O_22_, barium nitrate Ba(NO_3_)_2_, iron nitrate Fe(NO_3_)_3_·9H_2_O, glycine C_2_H_5_NO_2_, urea CON_2_H_4_, triethanolamine (TEA) C_6_H_15_NO_3,_ ethylene diamine tetraacetic acid (EDTA) C_10_H_16_N_2_O_8,_ glucose C_6_H_12_O_6_, ammonium hydroxide NH_4_OH, distilled water, Congo red dye C_32_H_22_N_6_Na_2_O_6_S_2_, ascorbic acid C_6_H_8_O_6_, isopropyl alcohol C_3_H_8_O, nitric acid HNO_3_, and sodium nitrate NaNO_3_ were obtained from Sigma-Aldrich, Germany; all used chemicals were analytical-grade chemicals.

### 2.2. Synthesis of Bi_0.9_Ba_0.1_FeO_3_ nanocomposites

**Fig. 2:**
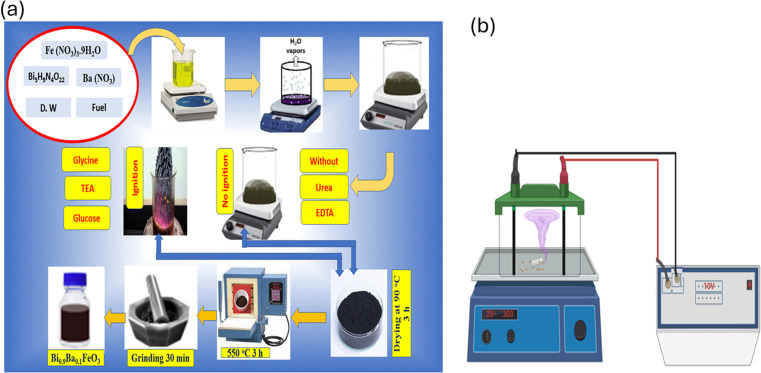
(a) Scheme of the co-precipitation processes of the nano-Bi_0.9_Ba_0.1_FeO_3_ and (b) electrochemical system for degradation of CR dye.

Nano-sized Bi_0.9_Ba_0.1_FeO_3_ powders have been synthesized by the combustion route; bismuth subnitrate, barium nitrate, and iron nitrate are mixed with an amount of fuel. Stoichiometric amounts of Bi_5_H_9_N_4_O_22_, Ba(NO_3_)_2_, Fe(NO_3_)_3_·9H_2_O were dissolved separately in distilled water to form the nitrate solutions (except Bismuth subnitrate, which was dissolved in a small amount of dilute nitric acid with distilled water). All solutions were mixed in one beaker and put on magnetic stirring at 600 rpm. The required amount of fuel glycine, urea, TEA, C_6_H_15_NO_3,_ EDTA, and glucose with a molar ratio determined according to the oxidation/reduction number of reactants in eqn (1), represented in Table 1, was added to the nitrates solution to form the precursor solution for Bi_0.9_Ba_0.1_FeO_3_ composition according to eqn (2). MI : TEA = 1 : 4, there is no need to adjust the pH for glycine, urea, and TEA. A molar ratio (MI : EDTA) = (2 : 1), the amount of EDTA is dissolved in distilled water with the addition of NH_4_OH until it reached a pH of 8–10. The nitrate solution was added dropwise to the EDTA solution to prevent an irreversible reaction. Glucose (12 g) was dissolved in 100 mL distilled water and added to the nitrate solution, keeping pH = 7 by carefully adding NH_4_OH. The precursor solution was heated on a hot plate with magnetic stirring at 600 rpm at 90 °C for 10 h, and the water gradually vaporized during heating and formed a transparent viscous gel. The flow chart is shown in Fig. 2(a).

**Table 1:** Structure parameters of Bi_0.9_Ba_0.1_FeO_3_ NPs

Parameter	EDTA	Glucose	Glycine	TEA	Urea	Without
Chemical formula	C_10_H_16_N_2_O_8_	C_6_H_12_O_6_	C_2_H_5_NO_2_	C_6_H_15_NO_3_	CON_2_H_4_	C_2_H_5_NO_2_
Reduction no.	40	24	9	33	6	9
*T* (combustion), ^o^C	280 flaming	200 flaming	180 smoldering	180 smoldering	250 smoldering	280 smoldering
% Perovskite phase	100	100	100	74	83	78
*a*, Å	3.9677	3.9337	3.9526	3.9503	3.9468	3.9783
*C*, Å	15.8311	15.7995	15.5289	15.8979	15.8563	15.4559
*V*, Å^3^	249.2237	244.4821	242.6094	248.0859	246.9972	244.6185
Cs, nm	26.66	23.96	23.78	18.55	18.52	27.66
W–H, nm	23.83	23.83	23.83	34.29	23.83	34.29
Grain size, nm	79.74	90.13	292.8	104.18	56.4	53.77
Dislocation × 10^−3^, nm^−2^	1.40	1.76	1.76	2.9	2.9	1.3
Strain *ε* × 10^−3^	6.15	5.2	6	7.03	7.47	5.22

For instance, glycine (5.4495 g) was used as fuel with and without solvent, as revealed in eqn (1).1
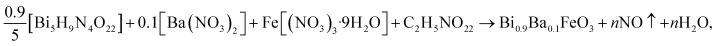
2
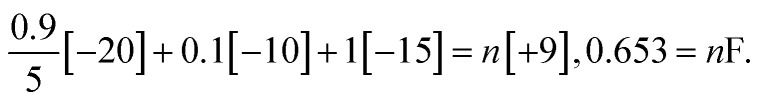


The total oxidizing and reducing valences of the fuel (F) and oxidizer (O) were used to calculate the stoichiometry of the redox mixture for combustion. This allowed the equivalency ratio, or *Φ*e (O/F), to reach unity and generate the maximum amount of heat. The glycine-to-nitrate ratio (GNR) was calculated based on the oxidizing and reducing valences of nitrates and glycine, respectively. The oxidizing valence of bismuth subnitrate is as follows:[Bi_5_H_9_N_4_O_22_ = 5 × 3 + 9 × 1 + 4 × 0 + (−2 × 22) = −20], [Ba(NO_3_)_2_ = 1 × 2 + 0 × 2 + (−2 × 6) = −10] and [Fe(NO_3_)_3_ = 1 × 3 + 0 × 3+ (−2 × 9) = −15],where the oxidizing valence of water is H_2_O = 0, and the reducing valence of glycine is [C_2_H_5_NO_2_ = 2 × 4 + 1 × 5 + 0 × 1+ (−2 × 2) = 9].

Thus, the total values of all nitrates should be balanced by the reducing valences of the fuel glycine. Therefore, eqn (2) states that *n* moles of fuel are needed to achieve the stoichiometric composition of the redox mixture, where *n* is the number of moles of fuel needed to release the most energy, and *F* is the total valences of fuel (equal to 9). Therefore, the appropriate moles of glycine, as shown in Table 1, are necessary for the stoichiometric composition of the redox mixture. Based on the abovementioned computation, stoichiometric reactant amounts were combined in an agate mortar and pestle. Because metal nitrates are hygroscopic, a slurry was created. A large, smoldering, fluffy product was produced in the beaker because the combustion process began with the slurry being dried at 80 °C and then heated to 150 °C.

### 2.3. Electrocatalytic measurements

A two-electrode electrochemical cell with a DC power supply voltage of 10 volts was used for the electrochemical reaction. Two electrodes were made of graphite, and 0.01 g of Bi_0.9_Ba_0.1_FeO_3_ catalyst was added to 200 mL of an aqueous solution of Congo red (CR: 10 ppm) dye before 10 mL of 1 M NaCl was added. An electrocatalytic technique was used to break down the CR dye in suspensions before they were exposed to radiation for 30 min, as shown in Fig. 2(b). The electrodes were spaced 2 cm apart. A UV-vis spectrophotometer measured the CR dye maximum absorption peak spectra in 3 mL of solution at various time points. The degradation percentage was evaluated using eqn (3).^53^3
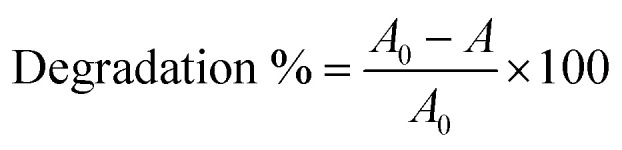
As a result, eqn (4) can be used to obtain the kinetic constant.4
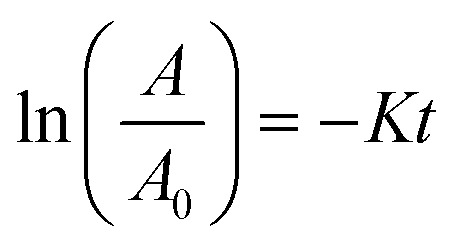
where *A*_0_, *A*, and *K* represent the initial absorbance, the absorbance at the time points during the degradation process, and the kinetic constant (min^−1^), respectively.

### 2.4. Devices and instrumental characterization

Phase identification and structure analysis of samples were investigated by XRD (Bruker, Germany) with CuK_α1_ radiation (*λ* = 1.5405 Å), and the step scan mode 2*θ* ranging from 10–80°, with a step size of 0.02°. XRD peaks were indexed using the X'pert High Score software program and Rietveld refinement software programs. FTIR (PerkinElmer Spectrum, Germany) spectroscopy was used to identify the functional groups in the *ν* 400–4000 cm^−1^ wavenumber range in the as-synthesized samples. The morphological and elemental analyses of synthesized samples were examined using SEM (JEOL JSM-6390, Japan), close-fitting with an energy dispersive spectroscopy (EDS) at a voltage of 15 kV and with different resolutions of 10 μm and 300 nm. The average particle size was calculated by using ImageJ software program. To perform DC measurements, the powder was pressed at 250 bars in a pellet shape with a 13 mm diameter and 2 mm thickness. A KEITHLEY cell station (616 digital electrometer, Germany) was used to measure the DC conductivity of prepared samples and DC electrical conductivity. A homemade electrometer device with a temperature range of ambient temperature to 150 °C in steps of 5 °C was used to measure the temperature dependency of the samples' DC conductivity. Before measurements, in the specifically made holder made of brass and Teflon as a two-probe contact, two copper plates were positioned between the samples as an ohmic contact. The vibrating sample magnetometer, VSM (Lake Shore-7410, Germany), was used to measure the magnetizations of all samples at room temperature within the magnetic field range of ±20 kOe.

## 3 Result and discussion

### 3.1. XRD analysis

**Fig. 3:**
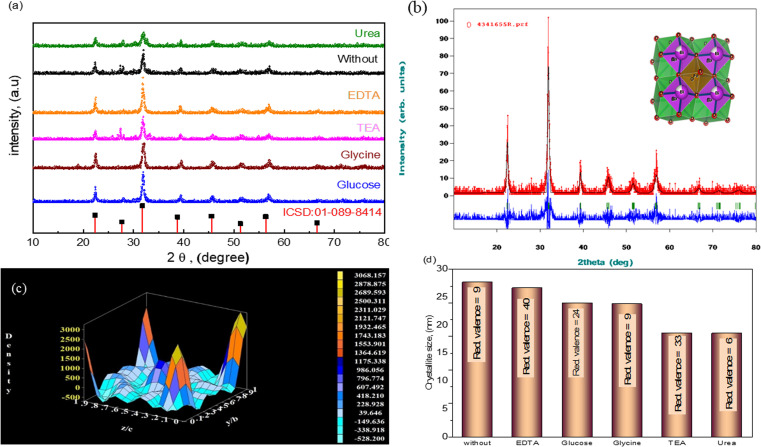
(a) XRD data of the Bi_0.9_Ba_0.1_FeO_3_ nanoparticles with different fuels, (b) Rietveld refinement using FullProf software of Bi_0.9_Ba_0.1_FeO_3_ nanoparticles, (c) X-ray electron density, and (d) crystallite size *vs.* solvent.

As shown in Fig. 3(a), the creation of a single-phase distorted tetragonal structure was demonstrated by the produced nanocomposites' XRD pattern with standard data (JCPDS #01-089-8414), which is described in the *P*4/*mmm* space group. The increase of Ba doping in such a composition transforms that structure to a cubic structure, whereas Bi_0.9_Ba_0.05_Sm_0.05_FeO_3_ nanocrystalline film has demonstrated a pure rhombohedral crystal structure, where BiFeO_3_, Bi_0.9_Ba_0.1_FeO_3_, and Bi_0.9_Sm_0.1_FeO_3_ films displayed a polycrystalline distorted rhombohedral crystal structure and belong to the *R*3*c* space group.^54,55^ The shift is caused by a minor difference in the atomic radii, and the indicated peaks of Bi_0.9_Ba_0.1_FeO_3_ correspond. The tiny change in the crystalline size of the nanocomposites is caused by the size difference in the ionic radius of the Bi^3+^, Ba^2+^, and Fe^3+^ ions linked to the compressive strain produced in the crystal matrix. The high-intensity peak of (014) at 31.9° appears as an overlapped single peak rather than peak splitting at *I*_max_, as is frequently observed in BFO nanoparticles, as shown in Fig. 3(a). It is noteworthy that the multiferroic samples exhibit a discernible drop in the intensity of the (014) peak, which could be caused by strain and the difference in atomic radii. This may impact the ferromagnetic and ferroelectric characteristics of BFO and doped BFO systems, particularly their functional behavior. Scherer's equation determined the crystallographic size based on the most noticeable peak at 2*θ* = 31.9°. According to eqn (5), the crystallite size (*D*) was calculated using the Debye–Scherrer formula:^56^5
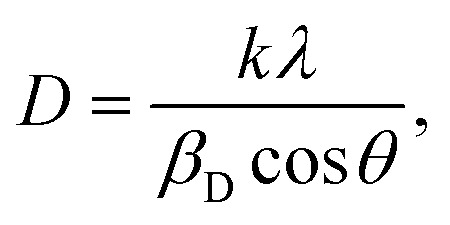
where *λ* is the incident radiation wavelength (1.5415 Å), *β*_D_ is the full-width half-maximum in radians, *θ* is the angle of diffraction, and *D* is the crystallite size of the most intense peak. The Scherrer equation yields a crystallite size from 26.66 to 18.52 nm, as shown in Fig. 3(d). XRD determines additional parameters, *e.g.*, the microstrain (*ε*) and the dislocation density (*δ*). Table 1 displays the unit cell's volume and lattice characteristics. The Williamson–Hall equation in eqn (6) is used to calculate the size of crystallites:6
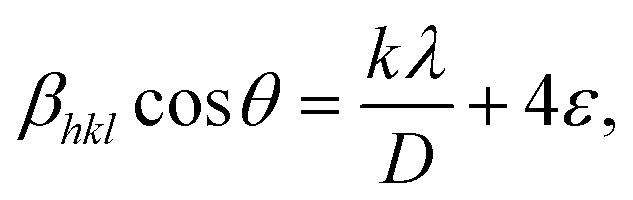


The dislocation density, using the square of the reciprocal of the crystallite size, was calculated using eqn (7).^57^7
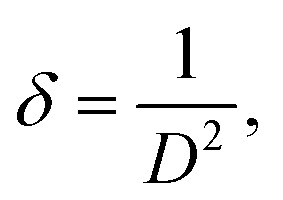


**Fig. 4:**
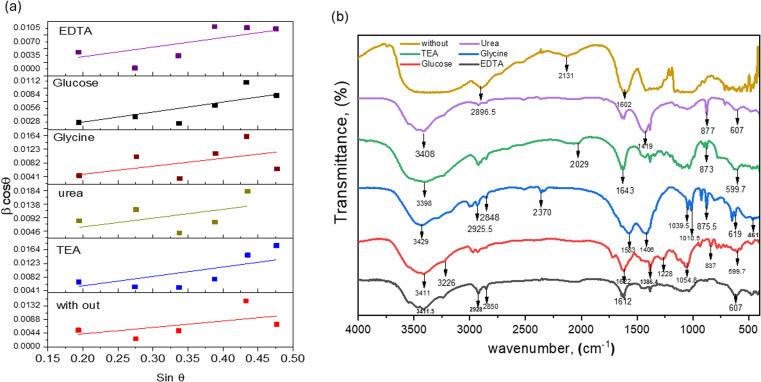
(a) W–H for Bi_0.9_Ba_0.1_FeO_3_ with different fuels and (b) FTIR spectra of Bi_0.9_Ba_0.1_FeO_3_ with different fuels.

The grasped microstrain is shown by the slope of the line (slope = 4*ε*), as shown in Fig. 4(a). The clear intersection of the line with the vertical *y*-axis confirms the crystallite size. This relationship, known as the Williamson–Hall relation, exists between *β* cos *θ* and sin *θ* [24]. The tolerance factor of Bi_0.9_Ba_0.1_FeO_3_'s unit cell calculations shows that it agrees well with the material's average crystalline size. Nonetheless, it was observed that several composites had a trace quantity of the Bi_24_Fe_2_O_39_ and Bi_2_O_4_ impurity phases. Bi_0.9_Ba_0.1_FeO_3_'s lattice parameters are similar to those in literature.^36^ Ba–O = 2.854 Å, Bi–O = 2.426 Å, and Fe–O = 2.028 Å are the bond lengths and atomic locations of different atoms taken from the Rietveld refinement of XRD data. Fig. 3(b) displays the crystal structures of Bi_0.9_Ba_0.1_FeO_3_ nanoparticles as determined by VESTA software. Fe atoms or cations are found at site 3b, Ba- and Bi-atoms or ions are found at site 3a, and oxygen is found at site 9e of the Wyckoff position. Six oxygen atoms encircle iron (Fe-) to create the octahedral structure of FeO_6_. The electrons in Fig. 3(d) have the same units as the F(H) per volume Fourier complex. Furthermore, many electrons are present at the same iron atomic site based on the electron distribution inside the unit cell. The electron density map inside our sample's unit cell is similar to the compound of Nd_0.5_Ba_0.5_FeO_3_.^58^

### 3.2. FTIR analysis

The FTIR spectra of Bi_0.9_Ba_0.1_FeO_3_, synthesized using the combustion method using different fuels, are shown in Fig. 4(b). The weak bands around *ν* 600 and 850 cm^−1^ are associated with the vibrational modes of the metal–oxygen bond: Fe–O stretching and the symmetric Bi/Ba–O/Fe–O vibrations in the BiBaFeO_3_ units, respectively.^59^ The stretching mode of the C–O bond is associated with the band at *ν* 1058 cm^−1^.^59^ As is well known, the chemisorption of ambient carbon dioxide results in the production of the carbonate group, which is represented by the band at *ν* 1406 cm^−1^.^59^ In certain compositions, the peak at *ν* 1372 cm^−1^ is assigned to the strong vibration of the NO^−^_3_ group. The peaks corresponding to the C–H stretching modes appear at *ν* 2920 cm^−1^. The hydroxyl groups at the low-coordination sites or faults cause the wide band at *ν* 3400 cm^−1^.^59^ However, doping with Ba ions broadens the band and the aforementioned functional groups of Bi_0.9_Ba_0.1_FeO_3_ (*ν* 522, 844, and 1365 cm^−1^, respectively).

### 3.3. SEM investigation

**Fig. 5:**
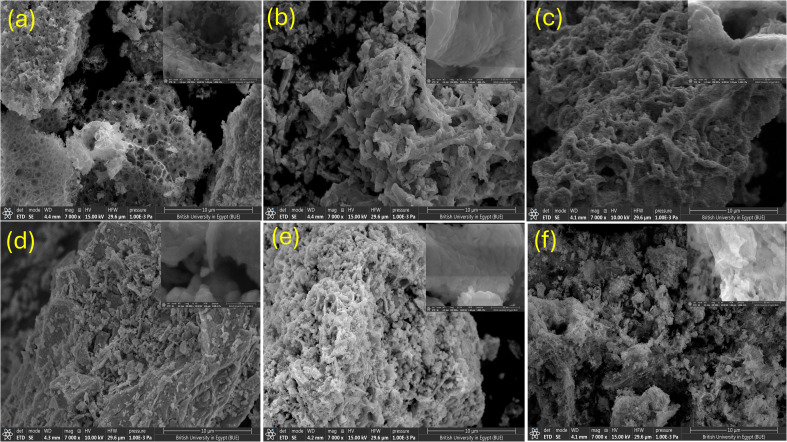
SEM of Bi_0.9_Ba_0.1_FeO_3_ at 10 micro-magnifications with different fuels: (a) EDTA, (b) glycine, (c) glucose, (d) urea, (e) TEA, and (f) without-fuel.

The nanostructure/morphology and elemental concentrations of Bi_0.9_Ba_0.1_FeO_3_ were determined using SEM/EDS investigations. Fig. 5 displays SEM micrographs of the synthesized nanomaterials. Bi_0.9_Ba_0.1_FeO_3_ nanoparticles' uneven morphology with aggregation was visible in the SEM images. For these tiny particles, the comparatively high magnetism caused the agglomerated particles to form. The high surface energy of the produced nanoparticles is thought to cause agglomeration. Due to doping and size reduction of the composites, this results in the unit-cell disorder. Herein, various Bi_0.9_Ba_0.1_FeO_3_ morphologies, including spongy structures with numerous cavities, coral-like, sheet-like, and rod-like forms, were synthesized using solvents such as EDTA, glucose-glycine, TEA, urea, and without any solvent, respectively. Such trends prove that the morphology of our samples depends not only on the synthesis method but also on the type of solvent used. Furthermore, the two combustion approaches, flaming or smoldering, affect the grain growth size, where the medium grain size forms in the flaming case. By contrast, the smoldering case creates agglomerated grain at low temperatures <200 °C and small grain at high temperatures >250 °C, as listed in Table 1.

**Fig. 6:**
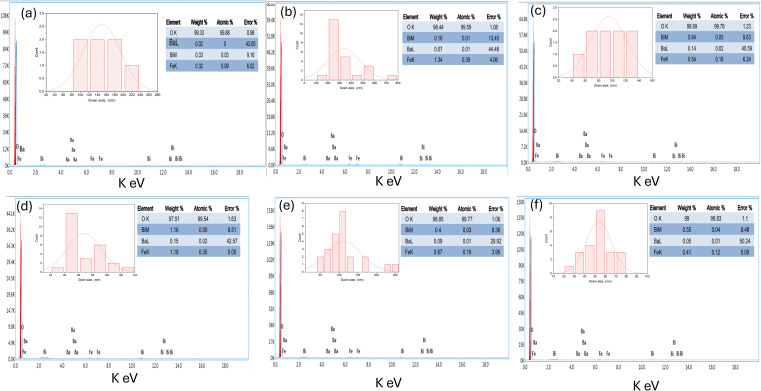
EDX and percentage of elements, and histogram of particle size of Bi_0.9_Ba_0.1_FeO_3_ NPs with different fuels: (a) EDTA, (b) glycine, (c) glucose, (d) urea, (e) TEA, and (f) without-fuel.

The energy dispersive spectrum (EDS) of Bi_0.9_Ba_0.1_FeO_3_ nanocomposites was presented in Fig. 6. It confirmed the presence of Bi, Ba, Fe, and O in our samples. No foreign elements in Bi_0.9_Ba_0.1_FeO_3_ were observed in Fig. 6. The agglomeration process and variety in grain morphologies are caused by irregular or discontinuous grain development, where some grains expand more quickly than others as the sintering temperature rises because many solvents are present.

### 3.4. DC conductivity measurements

**Fig. 7:**
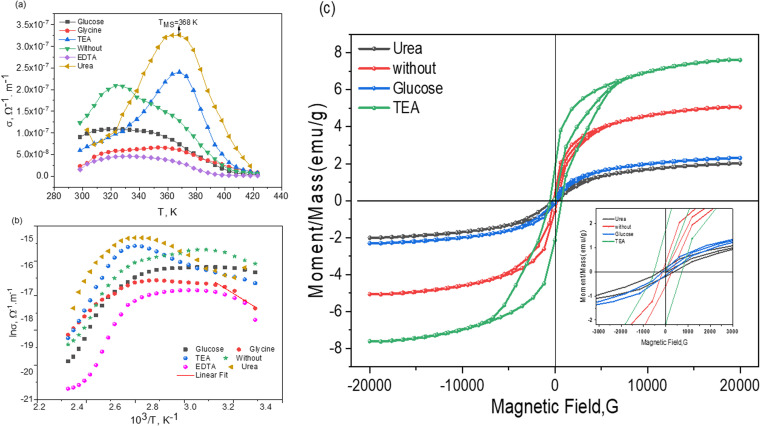
(a and b) DC conductivity dependence on temperature and (c) magnetization *vs.* magnetic field at room temperature for Bi_0.9_Ba_0.1_FeO_3_ NPs with different fuels.

The variation in DC conductivity (*σ*_DC_) of multiferroic nanoparticles as a function of absolute temperature is typical and shows an inverse relationship, as displayed in Fig. 7(a and b). Up to a crucial temperature *D*/2 (*D*: Debye temperature), a linear temperature dependency is evident.^60^ The DC conductivity then obeys the Arrhenius relation eqn (8), as the slope varies with divergence from linearity, and the activation energy is temperature-dependent. The DC electrical conductivity for samples was calculated according to the well-known Arrhenius equation as follows.^61,62^

8 *σ*_DC_ = *σ*_o_e^−*E*_a_/*K*_B_*T*^,

where *σ*_o_ is the pre-exponential factor, *E*_a_ is the activation energy in eV for electrical conductivity, *K*_B_ is the Boltzmann's constant *K*_B_ = 8.6 × 10^−5^ eV K^−1^, and *T* is the absolute temperature. The activation energy was calculated to be 0.39, 0.38, 0.30, 0.18, 0.16, and 0.075 eV for EDTA, glycine, urea, without, TEA, and glucose, respectively. At high temperatures, conductivity is generated by a thermionic emission process in which carriers possess enough energy to cross the potential barrier. It is obvious that *σ*_dc_ increases with temperature, indicating the samples' semiconducting nature. Additionally, a transition temperature of 368 K was observed. This temperature shifted to lower values for other fuels. The reduction in crystallite size may contribute to the increased conductivity. However, grain boundary scattering affects the electrical conductivity. These grain boundaries serve as traps, ensnaring electrons and creating a potential barrier. Conduction electrons scatter when this barrier is present at the grain boundaries, reducing conductivity. Therefore, the observed increase in conductivity can be attributed to the reduced grain boundary scattering^63^ caused by the smaller crystallite size.^60^

### 3.5. VSM measurement

We used a vibrating sample magnetometer (VSM) to examine the magnetic order of the prepared bismuth ferrite samples at ambient temperature. Notably, significant hysteresis was observed for every sample, with limited values of the coercive field, remnant magnetization, and saturation magnetization, which are listed in Table 2. Fig. 7(b) displays the M(H) loop for Bi_0.9_Ba_0.1_FeO_3_ ferrite at 300 K. Weak ferromagnetic order was also observed for urea and glucose samples; however, clear ferromagnetic behavior was observed for no-fuel and TEA samples. All samples reach magnetization saturation below 20 kOe magnetic field. Due to their grain size constriction, which has been reported to partially disrupt the long-range spiral spin structure of bulk BFO,^64^ Bi_0.9_Ba_0.1_FeO_3_ nanostructures with typical dimensions below 62 nm can exhibit advantageous magnetic characteristics.

**Table 2:** Crystallite size *D*, saturation magnetization *M*_s_, coercivity *H*_c_, remanent magnetization *M*_r_, and sequence of Ba-doped bismuth ferrites at room temperature

Methods	*M*_s_, emu g^−1^	*H*_c_, (Oe)	Mr	Seq. × 10^−3^	Ref.
Without solvent	5.0698	210.29	0.4923	97	Present work
TEA	7.6241	623.09	1.9590	257	
Glucose	2.3050	168.87	0.1262	55	
Urea	2.0105	325.30	0.1799	89	
Bi_0.9_Ba_0.1_FeO_3_, co-precipitation	1.46	50	—	—	54
Bi_0.9_Ba_0.1_FeO_3_, solid-state reaction	0.12	907 Oe	—	—	68
Bi_0.9_Ba_0.1_FeO_3_, sono-chemical technique	0.31 (at 8 kOe)	1918	0.04	—	64
Bi_0.9_Ba_0.1_FeO_3_, hydrothermal	7.24 (at 16 kOe)	2240	—	—	64
Bi_0.9_Ba_0.1_FeO_3_, co-precipitation	1.46	50	—	—	64
Bi_0.9_Ba_0.05_Sm_0.05_FeO_3_, Co-precipitation	0.15	200	—	—	64

The value of saturation magnetization, *M*_s_, comes out to be 2.01, 2.30, 5.06, and 7.62 emu g^−1^ for urea, glucose, no-fuel, and TEA, respectively, which is greater than the same composition value 0.31 emu g^−1^.^64^ Different shape anisotropy, magnetocrystalline anisotropy, and varying degrees of defects in the different nanoforms could cause this variation in *M*_s_ magnitude for our samples. The hysteresis loop's crossings with the vertical magnetization axis allow for extracting the remanence magnitude *M*_r_. The coercivity field, *H*_c_, can reflect the coercivity of a ferromagnet or ferrimagnet. The magnetic field intensity needed to bring the magnetic sample's magnetization down to zero once saturation has been attained is denoted by the value of *H*_c_. The Hc values obtained for our samples (623.09–168.87 Oe) are significantly higher than those reported in the literature. TEA sample has the greatest values of *M*_S_ (7.63 emu g^−1^), *M*_r_ (1.96 emu g^−1^), and coercive field *H*_c_ (623.09 Oe). This might be because their samples contain a secondary phase while others do not. As seen in the Table 2, magnetic properties, including coercive field, remnant magnetization, and saturation magnetization values, are similar to those previously reported in the literature.^65^ Since the ionic size of barium (1.42 Å) is larger than that of bismuth (1.03 Å), the variation in magnetic parameters for the same composition may be attributable to the incorporation (which greatly depends on the type of fuel) of Ba^3+^ in BFO may result in a significant off-center movement of Fe^3+^ ions in the octahedral.^66^ This kind of variation causes the perovskite structure to be distorted by doping, which results in a smaller Fe^3+^–O–Fe^3+^ bond angle and less antiferromagnetic interaction. As a result, the magnetization of Bi_0.9_Ba_0.1_FeO_3_ is significantly altered. Furthermore, barium has a valency of +2, while bismuth has a valency of +3. Therefore, charge compensation will be required for incorporating barium into the bismuth site. As a result, Fe^4+^ or oxygen vacancies may occur; the former may statistically distribute with Fe^3+^ in the octahedron in BBFO, resulting in ferromagnetism and net magnetization.^67^ Furthermore, BiFeO_3_ has a spiral spin magnetic structure with a period of 62 nm. Consequently, Bi_0.9_Ba_0.1_FeO_3_ nanograins that aggregate into ∼292 nm particles may fragment into grains that aggregate into ∼53 nm particles, resulting in improved magnetic properties.^66^

### 3.6. Electrocatalytic degradation of CR dye

**Fig. 8:**
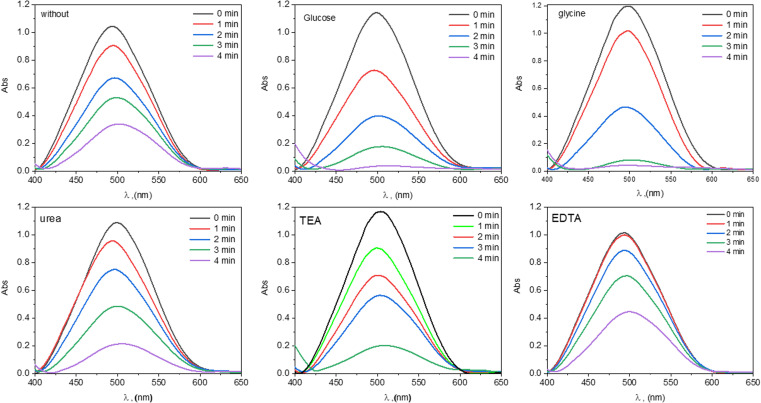
Absorption spectra of CR dye solutions under Bi_0.9_Ba_0.1_FeO_3_ NPs in different fuels.

The electrocatalytic activity of the prepared Bi_0.9_Ba_0.1_FeO_3_ samples was analyzed by studying the electro-degradation of CR dye (10 ppm) in aqueous solutions at constant voltage and at different time points. The absorption spectra of CR dye in aqueous solutions show an absorption band at 494 nm. These spectra were first recorded in the presence of electrocatalysts and at different irradiation times. However, when Bi_0.9_Ba_0.1_FeO_3_-based electrocatalysts are present, clear dye-degradation effects are visible. It is expected that the fuel type will have a significant impact on the catalytic activity of the nanoparticles, as shown by the absorption spectra for CR dye solutions in Fig. 8. The produced nanoparticles were utilized as a catalyst in the electrocatalytic oxidation process of CR dye, which was exposed to a constant voltage for up to 4 min, to examine the effects on the catalytic performance of the product particles from the reagent material used as fuel in the synthesis of Bi_0.9_Ba_0.1_FeO_3_ nanoparticles. The absorption band's intensity decreased as the irradiation period increased. Furthermore, compared to the solvent-free condition, the declining tendency was stronger for the solvent catalysts (Bi_0.9_Ba_0.1_FeO_3_). The breakdown of azo bonds in organic dyes demonstrates the synergistic effect of Fe- and Bi.^69^

**Fig. 9:**
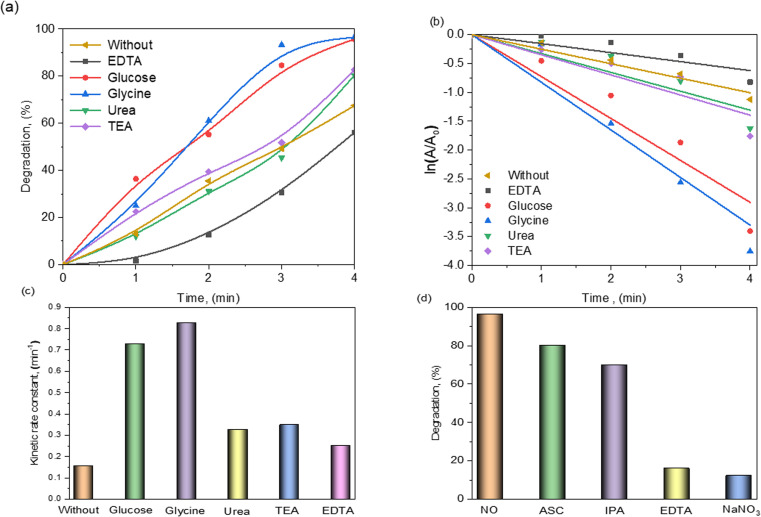
(a) Electro-degradation of CR dye against reaction time, (b) reaction rate, (c) variation of the kinetic coefficient of the electrocatalysis reaction, and (d) effect of radical scavengers on the electrocatalytic degradation of CR dye.

The effect of fuels on the structural and electrocatalytic properties of Bi_0.9_Ba_0.1_FeO_3_ NPs using glycine as a green fuel is investigated. The degradation of CR dye is shown in Fig. 9(a). Bi_0.9_Ba_0.1_FeO_3_ NPs achieved 67.4% degradation without fuel; similarly, the degradation increased up to 96.5% within 4 min in the presence of glycine, which is ascribed to its large grain size. The kinetics data are presented in Table 3. The determined rate constant of Bi_0.9_Ba_0.1_FeO_3_ NPs using glycine was 0.82486 min^−1^, which was four times higher than that for the sample prepared without fuel. Table 3 displays the regression correlation coefficient (*R*^2^) values, which were determined and ranged between 0.90 and 0.98. As seen from the *R*^2^ values, the reasonably excellent linear fits suggest that electro-degradation proceeds according to pseudo-first-order kinetics, as illustrated in Fig. 9(b and c). Bi_0.9_Ba_0.1_FeO_3_ NPs with glycine have a considerably smaller particle size compared with Bi_0.9_Ba_0.1_FeO_3_ particles without fuel and have significantly higher electrocatalytic activity. This is attributed to the available active sites on the particle's surface.

**Table 3:** Calculated degradation, pseudo-first-order rate constant (*k*), and regression correlation coefficient (*R*^2^)

Sample	Degradation (%)	Kinetic rate (min^−1^)	*R* ^2^
EDTA	56.03	0.15552	0.90782
Glucose	95.67	0.72607	0.96483
Glycine	96.50	0.82486	0.94641
Urea	80.34	0.34854	0.92737
TEA	82.68	0.01973	0.92938
No fuel	67.48	0.25147	0.98126

### 3.7. Influence of radical scavengers on electrocatalytic activity

Reactive species trapping studies are necessary to determine the mechanism underlying the high electrocatalytic activity of Bi_0.9_Ba_0.1_FeO_3_ electrocatalysts. The degradation (%) of CR dye in the presence of glycine of Bi_0.9_Ba_0.1_FeO_3_ NPs with EC was investigated using radical scavengers. Ascorbic acid (ASC), isopropyl alcohol (IPA), sodium nitrate NaNO_3_, and EDTA were used as scavengers of O_2_^−^˙, ˙OH, e^−^, and h^+^ in these assays.^70^ When CR dye was electrocatalytically broken down with EDTA present, the degradation efficiency dropped slightly from 96.5% to 15.9%, as illustrated in Fig. 9(d). Nevertheless, the degradation efficiency further decreased from 96.5% to 12.1% by adding NaNO_3_. This result indicates that the degradation of CR dye is caused by h^+^ and e^−^.

### 3.8. Electro-oxidation mechanism of CR

The overall catalysis of organic dye depends on its degradation in the presence of an electrocatalyst. The following method adds sodium chloride to CR dye during indirect electro-oxidation to improve conductivity, leading to the formation of hypochlorite ions.^71^

9 Anode: 2Cl^−^ → Cl_2_ + 2e^−^

10 Cathode: 2H_2_O + 2e^−^ → H_2_ + 2OH^−^

The Cl_2_ hydrolysis in the solution occurs following the subsequent reaction:

11 Cl_2_ + H_2_O ↔ HOCl + H^+^ + Cl^−^

and further dissociates generating a hypochlorite ion.

12 HOCl ↔ H^+^ + OCl^−^

In the electrocatalytic breakdown of CR dye effluent by chlorine production, the hypochlorite ions act as the primary oxidizing agent in pollutant degradation.

13 CR dye + OCl^−^ ↔ CO_2_ + H_2_O + Cl^−^

The valence band (VB) holes are produced when electrons from the valence band (VB) are driven into the conduction band after following an electric path longer than the band gap energy *E*_g_ of Bi_0.9_Ba_0.1_FeO_3_ NPs. Electrons travel through the external circuit to counter electrochemical potential during degradation, creating highly reactive superoxide anion radicals (O_2_).

14 e^−^ + O_2_ → O^−^_2_

15 h^+^ + H_2_O → OH˙

16 CR dye + OCl^−^ + OH˙→ CO_2_˙+˙H_2_O + Cl^−^

## 4 Conclusions

In the present work, the synthesis of Bi_0.9_Ba_0.1_FeO_3_ nanostructures using different fuels was achieved using the auto-combustion method. XRD reveals single-phase Bi_0.9_Ba_0.1_FeO_3_ nanomaterials alongside a small number of secondary phases. The R3C space group for the rhombohedral structure was assigned to samples with crystallite size ranging from 18.22 to 27 nm. SEM reveals that the grain size ranged from 56 to 292 nm. The change in magnetic behavior is due to the presence of smaller-sized nanoparticles. The highest values of *M*_S_ (7.63 emu g^−1^), *M*_r_ (1.96 emu g^−1^), and coercive field *H*_c_ (623.09 Oe) were observed in the TEA sample. This might be due to the secondary phase in that sample. DC electrical conductivity for samples was calculated according to the well-known Arrhenius equation, and the activation energy was estimated to range from 0.39 to 0.075 eV SPH, a behavior dominated by SPH in the high-temperature range, as indicated by the semiconductor behavior of the samples. Because of the effects on structural properties, the kind of fuel utilized is also extremely effective in influencing the catalytic activities of the nanoparticles, according to results of studies conducted to evaluate the electrocatalytic performances of Bi_0.9_Ba_0.1_FeO_3_. After the 4 min trial time, it was found that the CR dye concentration in the solution had nearly entirely deteriorated. When considered collectively and individually, the data show a clear correlation between the kind of fuel and crystallinity and, consequently, between crystallinity and electrocatalytic performance.

## Ethical statement

This article does not contain any studies related to animals performed by any of the authors.

## Data availability

The data supporting the findings of this article are included in the manuscript and are available upon reasonable request from the corresponding author.

## Author contributions

Mohamed Ghozza, Ahmed T. Mosleh: methodology, formal analysis, and data aquration. Tarek A. Yousef, Abdullah Al-Dakhil, Abeer M. Alosaimi, Reda Abdel-Hameed: resources, data analysis, and software: Elbadawy A. Kamoun, H. Zahran, Ibrahim S. Yahia: project management, supervision, wrote original-draft and reviewed the final draft. All authors approved the current and final version of manuscript for submission.

## Conflicts of interest

The authors declare that they have no known competing financial interests or personal relationships that could have appeared to influence the work reported in this paper.
